# Genistein-Supplemented Diet Decreases Malaria Liver Infection in Mice and Constitutes a Potential Prophylactic Strategy

**DOI:** 10.1371/journal.pone.0002732

**Published:** 2008-07-16

**Authors:** Margarida Cunha-Rodrigues, Sílvia Portugal, Miguel Prudêncio, Lígia A. Gonçalves, Cristina Casalou, Dominik Buger, Robert Sauerwein, Werner Haas, Maria M. Mota

**Affiliations:** 1 Unidade de Malária, Instituto de Medicina Molecular, Faculdade de Medicina da Universidade de Lisboa, Lisboa, Portugal; 2 Instituto Gulbênkian de Ciência, Oeiras, Portugal; 3 Angiogenesis Laboratory, Centro de Investigação em Patobiologia Molecular Instituto Português de Oncologia Francisco Gentil, Lisboa, Portugal; 4 DSM Nutritional Products, R&D Analytical Research Center, Basel, Switzerland; 5 Department of Medical Microbiology, University Medical Centre, Nijmegen, The Netherlands; 6 Alfama, Taguspark, Porto Salvo, Portugal; The Research Institute for Children at Children's Hospital New Orleans, United States of America

## Abstract

In tropical regions millions of people still live at risk of malaria infection. Indeed the emergence of resistance to chloroquine and other drugs in use in these areas reinforces the need to implement alternative prophylactic strategies. Genistein is a naturally occurring compound that is widely used as a food supplment and is thought to be effective in countering several pathologies. Results presented here show that genistein inhibits liver infection by the *Plasmodium* parasite, the causative agent of malaria. *In vitro*, genistein decreased the infection rates of both mouse and human hepatoma cells by inhibiting the early stages of the parasite's intracellular development. Oral or intraperitoneal administration of genistein decreased the liver parasite load of *P. berghei*-infected mice. Moreover, mice fed on a genistein-supplemented diet showed a significant reduction in *Plasmodium* liver infection as well as a reduced blood parasitemia and partial protection from severe disease. Since genistein is a safe, low-cost, natural compound that can be used permanently in a diet, we propose its use as a prophylactic agent against malaria for endemic populations and long-time travelers.

## Introduction

In recent years, *Plasmodium falciparum*, the most virulent malaria parasite species that infects humans, has developed increasing resistance to anti-malarial drugs in use. The development of new drugs has been slow and insufficient to overcome the problem of drug resistance. Moreover, the most affected populations in developing countries cannot afford most available therapies. In this context, not only toxicity but also cost effectiveness and distribution are important factors that need to be considered when developing novel intervention approaches against malaria.

The hepatic stage is the first step of any natural malaria infection and a prerequisite for the subsequent blood stage, when disease-associated pathology occurs. The obligatory, yet silent, nature of liver stage infection makes it an attractive target for prophylactic anti-malarial intervention strategies [1]. Moreover, a decrease of the parasite load in the liver has been shown to be associated with a decrease in the severity of the disease [2], [3]. Clinical manifestations of malaria cover a wide range of symptoms. Although most infected individuals will only have a relatively benign febrile illness, 1–3 million deaths per year occur from severe malaria, which includes several syndromes such as severe anaemia, acute respiratory distress or cerebral malaria.

Genistein, a major component of soybeans, is a broad tyrosine kinase inhibitor [4] and has been ingested by several Asian populations for centuries without any obvious adverse effects. Several clinical trials have successfully addressed the usefulness of genistein as a prophylactic agent for certain types of cancer and chronic diseases [5]. Moreover, genistein has been shown to inhibit *in vitro* the intraerythrocytic development of *P. falciparum* and *P. chabaudi*, an effect that has been attributed to its action as a tyrosine kinase inhibitor [6]–[8]. We have previously shown that, in the liver, signaling through the host tyrosine kinase receptor MET facilitates sporozoite infection [9]. Thus, we hypothesized that genistein might influence hepatic infection by *Plasmodium* and sought to investigate its potential as a prophylactic anti-malarial. Here, we demonstrate that genistein affects *P. berghei* ANKA sporozoite development within hepatocytes and thereby reduces hepatic infection both *in vitro* and *in vivo*. Importantly, we show that genistein given as a food supplement to mice affects the full course of a malaria infection, including the development of pathology, by reducing the extent of liver infection by *Plasmodium*.

## Results

### Genistein Inhibits *in vitro* Hepatocyte Infection by *P. berghei* ANKA Sporozoites

**Figure 1:**
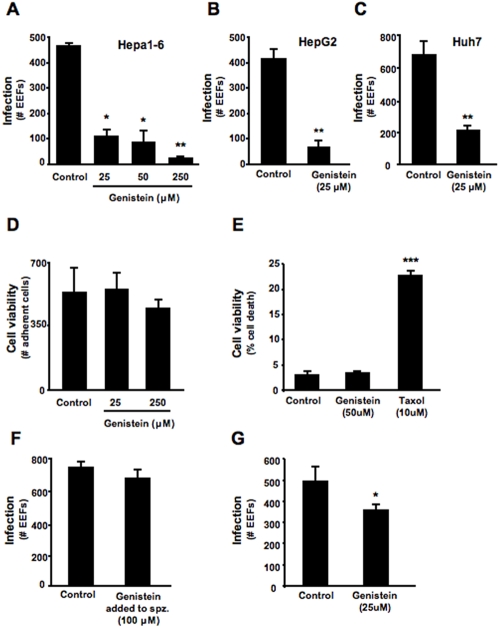
Genistein inhibits *in vitro* hepatoma cell infection by *Plasmodium* sporozoites. Cultured Hepa1-6 cells were incubated with increasing doses of genistein (A) and cultured HepG2 and Huh7 cells were incubated with 25 μM of genistein (B) and (C). As control, cells were treated with equivalent volumes of DMSO as in the genistein treated groups. The number of infected cells was determined 24 hours after infection with 4×10^4^ *P. berghei* ANKA sporozoites and is shown as the total number of EEFs in each coverslip. Each condition was assayed in duplicate in (A) or triplicate in (B) and (C). ★ p<0.05, ★★ p<0.01 (TTest relative to control group). Bar plot shows one representative of 3 independent experiments, error bars represent s.d. of mean number of EEFs in each condition. (D) Cultured HepG2 cells were incubated with different concentrations of genistein, or DMSO (control). After 24 hours the number of adherent cells in 10 microscope fields representing approximately 20% of the total area was assessed at 400× magnification. Each condition was assayed in duplicate. (E) Cultured Huh7 were incubated with Genstein or Taxol, along with their respective controls of DMSO, and allowed to grow for 24 hours. After this time both adherent and non-adherent cells were collected and incubated with propidium iodide. Percentage of death cells was quantified by flow cytometry. Bar plot shows one representative of 3 independent experiments, error bars represent s.d. of mean cell death percentage in each condition (n = 3). ★★ p<0.01 (TTest relative to control group). (F) *P. berghei* ANKA sporozoites were incubated with 100 μM genistein or DMSO for 30 min. Sporozoites were washed with PBS to remove genistein and used to infect cultured HepG2 cells. Infection was determined 24 hours post-infection by counting the total number of EEFs in each coverslip. Each experimental condition was assayed in triplicate. Bar plot shows one representative of 3 independent experiments. Error bars represent s.d. of mean number of EEFs in each condition (*n* = 3). (G) Huh7 cells were incubated with 25 μM genistein or DMSO for 2 h. Cells were then washed with PBS and fresh medium to remove genistein. P. berghei sporozoites were then added to these cells. Infection was determined 24 hours post-infection by counting the total number of EEFs in each coverslip. Each experimental condition was assayed in triplicate. Bar plot shows one representative of 3 independent experiments. Error bars represent s.d. of mean number of EEFs in each condition (*n* = 3).

**Figure 2:**
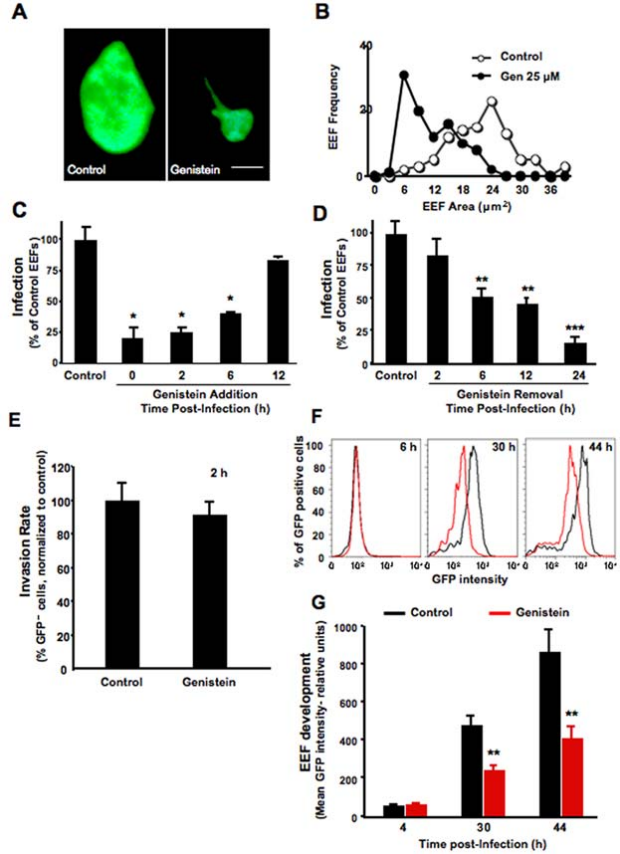
Genistein suppresses the early development of parasites within hepatocytes. (A) Hepa1-6 cells were incubated with genistein or DMSO and inoculated with 4×10^4^ *P. berghei* ANKA sporozoites. The two images show EEFs observed 24 hours after infection of control (left) or genistein-treated cells (right) at a 1000× magnification. Bar = 2.5 μm. (B) Distribution of EEFs according to size. Hepa1-6 cells were treated with genistein or DMSO (control) immediately prior to inoculation with 4×10^4^ *P. berghei* ANKA sporozoites and the size of 100 EEFs from different coverslips was determined 24 hours later. The figure shows the frequency of EEFs with sizes between 0 and 40 μm^2^. ○ control; • 25 μM genistein. *P* = 5.9×10^−^^25^ (TTest relative to the control group). The results are representative of 3 independent experiments. (C) Incubation of cultured HepG2 cells with 25 μM genistein or DMSO (control) at various times after addition of 4×10^4^ *P. berghei* ANKA sporozoites. Infection was determined 24 hours after sporozoite addition by counting the total number of EEFs per coverslip. The results are expressed as the percentage of EEFs relative to the average number of EEFs in the control samples, taken as 100%. (D) Incubation of cultured HepG2 cells with 25 μM genistein or DMSO (control) at the time of addition of 4×10^4^ *P. berghei* ANKA sporozoites and washed at various time-points. Infection was determined 24 hours after sporozoite addition by counting the total number of EEFs per coverslip. The results are expressed as the percentage of EEFs relative to the average number of EEFs in the control samples, taken as 100%. ★ p<0.05, ★★ p<0.01 ★★★ p<0.001 (TTest relative to control group). Bar plots show one representative of 3 independent experiments, error bars represent s.d. of mean number of EEFs in each condition (*n = *3). (E) Flow cytometry analysis of the invasion rate in genistein-treated and control Huh7 cells at 2 hours after addition of 3×10^4^ PbGFP sporozoites. Bar plots show one representative of 3 independent experiments, error bars represent s.d. of mean percentage of GFP positive cells in each condition (*n = *3). (F) Flow cytometry analysis of genistein-treated and control Huh7 cells at 6, 30 and 44 hours after addition of 3×10^4^ PbGFP sporozoites, and of primaquine-treated and control cells at 44 hours after adition of 3×10^4^ PbGFP sporozoites. Red line represents Genistein treated cells and black line represents control cells. The graphs show one representative dataset of triplicate samples. (G) Quantification of the GFP intensity of PbGFP-infected genistein-treated (red bars) and non-treated (black bars) cells at the same time points as in E. ★★ p<0.01 (TTest relative to control group). Bar plots show one representative of 3 independent experiments, error bars represent s.d. of mean GFP intensity in each condition (*n = *3).

To determine whether genistein has any effect on the hepatic infection by *Plasmodium* sporozoites, cultures of Hepa1-6 cells, a mouse hepatoma cell line, were infected *with P. berghei* ANKA sporozoites in the presence of different doses of genistein. Twenty-four hours after sporozoite addition, the number of infected cells was determined by counting the total number of intracellular developing parasites (exo-erythrocytic forms, EEFs). The results show that genistein significantly inhibits Hepa1-6 cell infection in a dose-dependent manner (p<0.05) (Figure 1A). We also observed that genistein decreases infection by *Plasmodium* sporozoites in 2 different human hepatoma cell lines, HepG2 or Huh7 cells (74±8% and 68±4% reduction in infection, respectively; p<0.01), to a similar extent as that observed in the murine cell line (Figure 1B, C). Genistein treatment does not lead to any signs of loss of cell viability or reduced proliferation. Indeed, HepG2 or Huh7 cells treated with genistein show constant number of cells as well as constant proportion of cell death (Figure 1D, E). Taxol treated Huh7 cells were used as positive control. To test the possibility that genistein has a direct effect on *Plasmodium* sporozoites, preventing them from invading the cells, *P. berghei* ANKA sporozoites were incubated with 100 μM genistein for 30 min and, after several washes to remove genistein, added to HepG2 cells. EEF number was assessed 24 hours later and no significant difference was observed between the infection levels of cells infected with sporozoites pre-treated with genistein and those infected with vehicle-treated sporozoites (Figure 1F). Interestingly, pre-treatment of cells for 2 h prior to sporozoite addition led to a small but significant reduction of infection, which again strongly suggests the effect of genistein in infection is due to an effect on the host cell and not on the parasite (p<0.05) (Figure 2G). Altogether the data shows that genistein strongly affects host cell infection by *P. berghei* ANKA without having a direct action on sporozoites.

### Genistein Impairs Early *Plasmodium* Sporozoite Development Inside Host Cells

After invasion of a hepatocyte, each parasite replicates to thousands of merozoites that are subsequently released into the blood stream. As parasites develop and replicate inside hepatocytes, the size of EEFs increases. We noticed a striking difference between the sizes of EEFs in control *versus* genistein-treated cells (Figure 2A). The quantification of EEF areas in both groups of cells showed that EEFs are significantly smaller in genistein-treated cells than in untreated controls (Figure 2B), suggesting that genistein affects the development of *Plasmodium* sporozoites inside host cells.

Hepatocyte infection by *Plasmodium* may be divided into three consecutive steps: cell traversal, productive hepatocyte invasion and intracellular development. In our *in vitro* infection model, over 95% of the infective sporozoites have completed the migration and invasion steps at 2 hours after addition to the cells [10]. Later, after 6 hours, sporozoites have changed their morphology and have acquired a round form and after 12 hours their size has started to increase. In order to identify the time-scale of genistein action, the drug was added to HepG2 cell cultures at different times after addition of *P. berghei* ANKA sporozoites. Genistein significantly reduced infection to approximately the same extent regardless of whether it was added to the cells prior to infection or up to 6 hours after sporozoite addition (p<0.05) (Figure 2C). This finding suggests that genistein reduces infection not by interfering with sporozoite invasion but rather by interfering with the development of sporozoites inside host cells. The effect of genistein was progressively lost when it was added at later time-points (Figure 2C). Still with the objective of finding genistein's time of action we added the drug to HepG2 cell cultures immediately prior to addition of *P. berghei* ANKA sporozoites, and then removed it at different times after infection (Figure 2D). Genistein reduced infection when it was present during the first 4 hours of infection. The degree of inhibition increases with exposure time and is most significant when the drug is present during the whole course of infection (p<0.001) (Figure 2D).

To further examine the effect of genistein in the development of sporozoites inside host cells, we used a recently described Flow Activated Cell Sorting (FACS) method [10] where we showed that EEFs of *P. berghei* ANKA expressing GFP show GFP intensity proportional to the EEF developmental stage. Having shown that genistein-treated cells and their controls were equally invaded by *P. berghei* ANKA expressing GFP sporozoites at 2 hours post–infection (Figure 2E), genistein-treated cells were collected and analyzed by flow cytometry at 6, 30 and 44 hours, after addition of *P. berghei* ANKA expressing GFP sporozoites. Solvent-treated cells infected with *P. berghei* ANKA expressing GFP sporozoites, collected and analyzed at the same times, were used as negative controls. Whereas the GFP intensity of infected and solvent-treated cells increases significantly with time as a result of the parasites' intra-cellular development, the increase in GFP intensity of genistein-treated cells is significantly less pronounced (Figure 2F). The quantification of the GFP intensity of triplicate samples of genistein-treated and control cells at each of the time-points analyzed shows that in the first hours after infection both sets of samples show similar GFP intensities, and the difference between these intensities then increases with time (p<0.01) (Figure 2G). These results provide evidence that genistein acts subsequent to cell invasion, by inhibiting the parasites' early development inside host cells.

**Figure 3:**
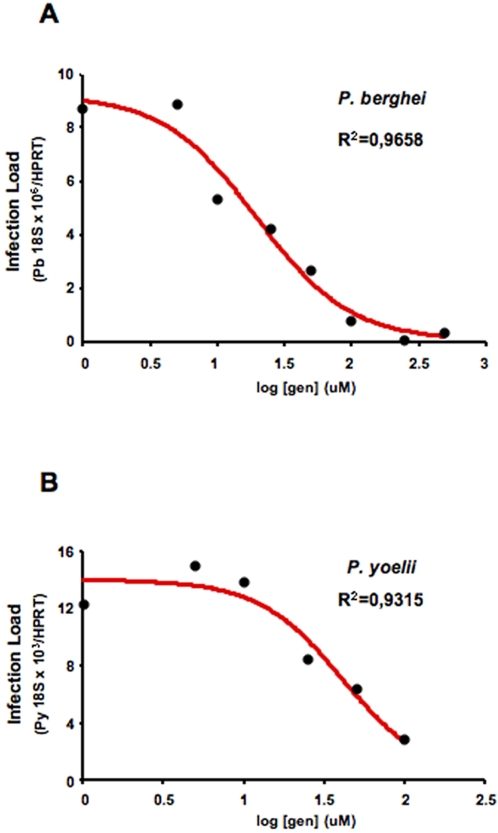
Genistein's 50% Inhibitory Concentration (IC50). (A) Cultured Huh7 cells were treated with increasing doses of genistein or DMSO (control), and inoculated with 4×10^4^ *P. berghei* ANKA sporozoites. Infection was determined 24 hours later by parasite-specific 18S rRNA qRT PCR, and an IC50 of 20 μM was calculated for genistein's inhibition of infection. Black circles represent the mean of *P. berghei* ANKA18S rRNA expression in each condition (*n = *3). (B) Cultured Hepa1-6 cells were treated with increasing doses of genistein or DMSO (control), and inoculated with 3.5×10^4^ *P. yoelii* sporozoites. Infection was determined 24 hours later by parasite-specific 18S rRNA qRT PCR, and an IC50 of 30 μM was calculated for genistein's inhibition of infection. Black circles represent the mean of *P. berghei* ANKA18S rRNA expression in each condition (*n = *3).

The concentration of genistein required to inhibit infection by 50% (IC50) was determined for *P. berghei* ANKA infection of Huh7 cells (p<0.05) (Figure 3A), and for *P. yoelii* infection in Hepa1-6 cells (p<0.05) (Figure 3B). *Plasmodium* infection of cells treated with increasing amounts of genistein was measured by quantitative real-time PCR (qRT-PCR), as this method takes into account both the number and the development of EEFs, since both contribute to the total number of parasite copies detected. Genistein inhibited infection with an IC50 of ∼20 and ∼30 μM for *P. berghei* and *P. yoelii* infections, respectively. This was performed in parallel to experiments where primaquine was tested in *P. berghei* infection of Huh7 cells. The IC50 value obtained for primaquine in those conditions was 13 μM (with an R^2^ value of 0.9658). In addition to hepatoma cell lines primary mouse hepatocyte cultures were also used. As observed in the cell lines, genistein reduced the infection of these cells by *P. berghei* sporozoites (data not shown). Our findings demonstrate that genistein impairs the developmental/replication of the parasite in cultured hepatocytes without affecting the viability of the host cells.

### Genistein Decreases *in vivo* Mouse Liver Infection by *P. berghei* ANKA Sporozoites

**Figure 4:**
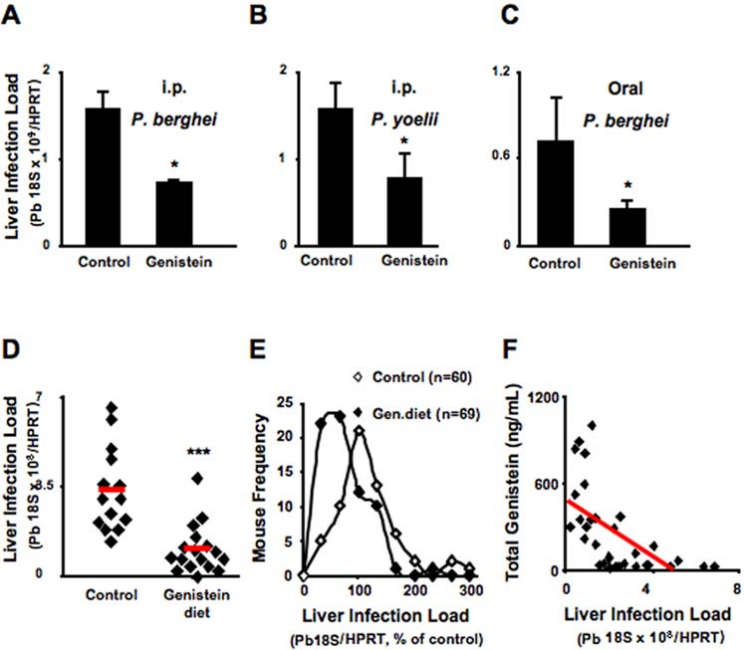
Genistein decreases mouse liver lnfection by *P. berghei* ANKA sporozoites. (A) Mice treated by intraperitoneal injection of 4 mg of genistein in a volume of 200μL DMSO, or with DMSO alone (control), were injected intravenously with *P. berghei* ANKA sporozoites (1×10^4^/mouse). Infection was determined 40 hours later by parasite-specific 18S rRNA qRT PCR (*n = *4 mice per group). ★ p<0.05 (TTest relative to control group). Bar plots show one representative of 3 independent experiments. Error bars represent s.d. of mean *P. berghei* ANKA 18S rRNA expression in each condition (*n = *3). (B) Mice treated by intraperitoneal injection of 4 mg of genistein in a volume of 200μL DMSO, or with DMSO alone (control), were injected intravenously with *P. yoelii* ANKA sporozoites (1×10^4^/mouse). Infection was determined 40 hours later by parasite-specific 18S rRNA qRT PCR (*n = *4 mice per group). ★ p<0.05 (TTest relative to control group). Bar plots show one representative of 3 independent experiments. Error bars represent s.d. of mean *P. yoelii* ANKA 18S rRNA expression in each condition (*n = *3). (C) Mice treatment by oral administration of 4 mg of genistein suspended in 200 μL water or with the same volume of water alone (control), 6 hours prior to intravenous injection of sporozoites (1×10^4^/mouse). Infection was determined 40 hours later by parasite-specific 18S rRNA qRT-PCR. *n = *3 mice per group. ★ p<0.05 (TTest relative to control group). Bar plots show one representative of 3 independent experiments. Error bars represent s.d. of mean *P. berghei* ANKA 18S rRNA expression in each condition (*n = *3). (D) Mice kept on a genistein-supplemented diet since breastfeeding or with the same diet without supplementation as a control, were injected intravenously with *P. berghei* ANKA sporozoites (1×10^4^/mouse). Infection was determined 40 hours later by parasite-specific 18S rRNA qRT-PCR. *n = *14 control group; *n = *17 genistein treated group. ♦ individual mouse, red bar represents the group average. *P* = 0.00007 (TTest relative to control group). Results are representative of 3 independent experiments. (E) Compiled data of liver infection from 5 independent experiments with mice fed on genistein-supplemented diet for a minimum of 5 weeks and injected intravenously with *P. berghei* ANKA sporozoites (1×10^4^/mouse). Infection was determined 40 hours later by parasite-specific 18S rRNA qRT-PCR. Results are expressed as frequency of mice that present a certain level of infection. Infection is expressed as the percentage of parasite specific 18S rRNA taking the average control (non-supplemented diet) as 100%. ⋄ control diet (*n = *60), ♦ 1000 ppm genistein-supplemented diet (*n = *69). *P* = 5.8×10^−^^5^ (Wilcoxon rank sum test to control group). (F) Inverse correlation between genistein levels in the sera and *Plasmodium* infection in the liver. Mice kept under genistein-supplemented diet since breastfeeding or with the same diet without supplementation, used as controls, were injected intravenously with *P. berghei* ANKA sporozoites (1×10^4^/mouse). Infection was determined 40 hours later by parasite-specific 18S rRNA qRT-PCR, sera were collected and genistein levels were determined for individual mice. Total genistein levels (ng/mL) in the sera are plotted against liver infection of individual mice. Infection is expressed as the percentage of parasite specific 18S rRNA taking the average control (non-supplemented diet) as 100%. ♦ represent the coordinates for genistein level (yy) and infection (xx) for individual mice. The red line represents the inverse correlation between the two parameters measured.

To investigate the *in vivo* relevance of our findings with cultured hepatocytes we treated mice with genistein by intraperitoneal (i.p.) administration immediately prior to the intravenous (i.v.) inoculation of *Plasmodium* sporozoites. Solvent-treated mice were used as controls. Quantification of parasite load in the livers 40 hours after *P. berghei* or *P. yoelii* sporozoite injection showed that a single i.p. administration of genistein at a dose of 200 mg/kg reduced infection (p<0.05) (Figure 4A, B). We then tested the effect of oral administration of a genistein suspension in water to mice prior to i.v. injection of *P. berghei* ANKA sporozoites. Water was administered to control mice. Administration of a single 200 mg/kg dose of genistein 6 hours prior to sporozoite inoculation, aiming to increase genistein levels in the liver at the time of sporozoite development, resulted in a 64% reduction of liver infection of genistein-treated mice relative to their control counterparts, measured 40 hours after infection (p<0.05) (Figure 4C).

The above findings constitute a proof-of-principle that genistein administered orally may be useful for malaria prophylaxis. However, prophylactic interventions based on daily drug administration are difficult to implement in developing countries, in particular if the whole population requires treatment. Thus, we sought to test the effect of genistein administration through diet. Mice were kept on a 1000 ppm genistein-supplemented diet since weaning (see methods for details), while control mice were kept on exactly the same diet but without genistein supplementation. Seven to 10 weeks old mice were inoculated i.v. with *P. berghei* ANKA sporozoites and liver infection was determined 40 hours after sporozoite injection. The liver parasite load was significantly reduced in mice kept on the genistein-supplemented diet relative to the untreated controls (p<0.001) (Figure 4, D and E). This effect was even more pronounced on the progeny of couples kept on this genistein-supplemented diet, presumably because those litters were exposed to genistein supplementation since breastfeeding. Moreover, the degree of infection was inversely correlated to the genistein levels in the sera of the mice (Figure 4F). These results show that the genistein levels achieved by a genistein-supplemented diet are sufficient to decrease the infection of the liver with *Plasmodium*.

### Genistein Decreases Disease Severity Through an Effect on Liver Stage Infection

**Figure 5:**
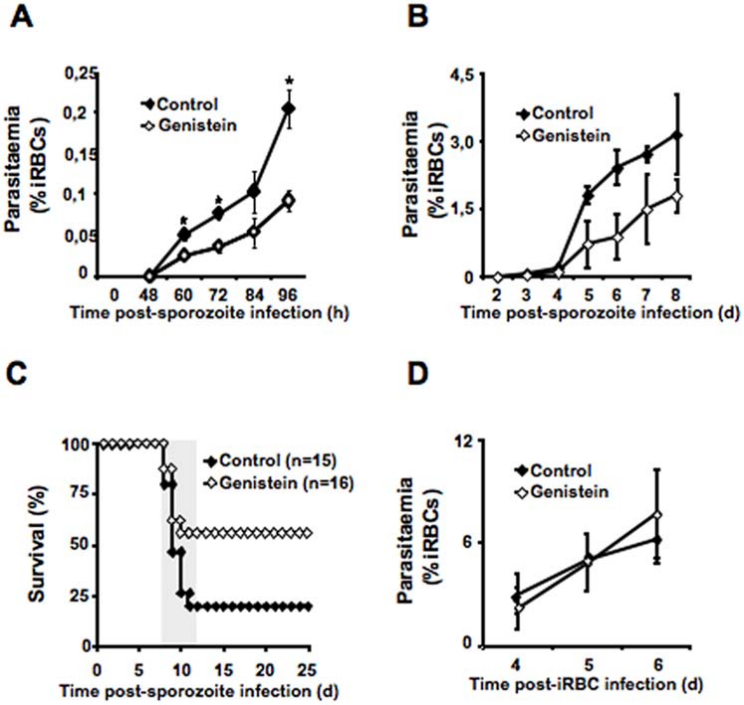
Genistein partially protects from cerebral malaria. (A) Mice fed on a genistein-supplemented or control diet since breastfeeding were injected intravenously with *P. berghei* ANKA sporozoites (1×10^4^/mouse) and maintained for the analysis of blood infection. Peripheral blood parasitemia was determined, at different time points after infection, by counting the number of infected red blood cells in Giemsa stained thin blood films. ♦ control diet; ⋄ 1000 ppm genistein-supplemented diet, *n = 3* mice per group. ★ p<0.005 (TTest relative to control group). The results are representative of 3 independent experiments with a total of 15 control mice and 19 genistein-treated mice. (B) Same as in A between day 2 and day 8 after intravenous injection of *P. berghei* ANKA sporozoites by counting the number of infected red blood cells in Giemsa stained thin blood films. ♦ control diet; ⋄ 1000ppm genistein-supplemented diet, *n = *3 mice per group. (C) Cumulative survival of mice fed on a genistein-supplemented or control diet since breastfeeding when injected intravenously with *P. berghei* ANKA sporozoites (1×10^4^/mouse). Fifty-six percent of the mice fed on the genistein-supplementd diet and infected with *P. berghei* ANKA sporozoites are protected from developing cerebral malaria when compared with twenty percent of mice fed on the control diet. ♦ control diet, *n = *15; ⋄ 1000 ppm genistein supplemented diet, *n = *16. The gray area represents the time window for developing cerebral malaria in this model. *P* = 0.067 (Log-Rank Test). (D) Mice fed on a genistein-supplemented or control diet since breastfeeding were infected by intraperitoneal injection of 1×10^6^ *P. berghei* ANKA iRBC. Peripheral blood parasitemia was determined throughout infection by counting the number of infected red blood cells in Giemsa stained thin blood films. Parasitemia indicates the percentage of iRBC in a given number of total red blood cells. ♦ control diet, *n = *6; ⋄ 1000 ppm genistein-supplemented diet, *n = *7. Results are representative of 2 independent experiments with a total of 11 control mice and 12 genistein-treated mice.

By reducing liver infection and, consequently, the number of merozoites that are generated in the liver, genistein treatment is likely to reduce the parasite burden in the blood. Thus, we determined whether the genistein diet had an impact on the blood stage of infection, which is responsible for disease symptoms. Mice kept on a genistein diet and controls were inoculated i.v. with *P. berghei* ANKA sporozoites and infection was allowed to proceed to the blood stage. Parasitemia (percentage of infected red blood cells in peripheral blood) was determined at regular time intervals. In genistein-fed mice the appearance of blood parasitemia was significantly reduced as compared to controls (p<0.01 for each day) (Figure 5A) and the percentage of parasitized red cells, remained lower throughout the entire course of the infection (Figure 5B). C57BL/6 mice infected with *P. berghei* ANKA die within 6–12 days due to the development of a complex neurological syndrome consisting of hemi- or paraplegia, head deviation, tendency to roll–over on stimulation, ataxia and convulsions. Given its similarities to human CM, this neurological syndrome is referred to as experimental cerebral malaria (ECM). This model has been used for some time by several different groups around the world. We have previously used this model and have observed that the mice present blood-brain-barrier disruption as soon as symptoms arise [11]. Importantly, more than half of the mice fed on the genistein-supplemented diet were protected from developing ECM. At 25 days after infection, the survival of mice fed on the genistein-supplemented diet and that of mice fed on a control diet without genistein supplementation was 56% and 18%, respectively (p<0.001) (Figure 5C). The reduction in blood infection could be due to the observed effect on liver infection but also to a direct effect of genistein on the blood stages. To test the latter possibility, mice fed on a genistein-supplemented diet were infected with *P. berghei* ANKA-infected red blood cells (iRBC) and their parasitemias were compared to those of untreated, infected mice. There was no significant difference between the development of parasitemia in both groups of mice (Figure 5D). These results show that, by affecting the liver stage of infection, genistein reduces the parasite load in the blood and the severity of the disease.

### Genistein Inhibits Sporozoite Induced Met Phosphorylation

**Figure 6:**
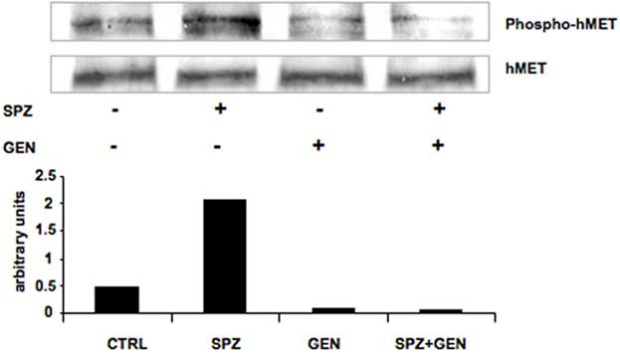
Effect of genistein on *Plasmodium* sporozoite-induced MET phoshorylation. Cultured Huh7 cells were treated with genistein or DMSO (control), and inoculated with 3×10^4^ *P. berghei* ANKA sporozoites. Four hours later total protein extracts were analyzed with anti-phospho c-met (Tyr1234/1235) and MET antibodies. Quantification of intensity of each band was performed and plotted on a graph.

The rationale to test the effect of genistein, a tyrosine kinase inhibitor, in infection was that signaling trough the tyrosine kinase receptor Met might favor parasite development in hepatocytes. To determine whether genistein inhibited parasite-induced Met phosphorylation genistein or DMSO was added to cultured Huh7 cells and the cultures were inoculated with 3×10^4^ *P. berghei* ANKA sporozoites. Four hours later total protein extracts were analysed with anti-phospho c-met (Tyr1234/1235) and MET antibodies. As shown in Figure 6, sporozoite infection induced Met phosphorylation and this was inhibited by genistein.

## Discussion

Prophylaxis against malaria remains a major concern in regions where the disease is endemic. Existing regimens for malaria prophylaxis are suitable for short time travelers but not for populations living in endemic areas. An alternative to most current strategies to combat malaria is to target and modulate host components known to be involved in the establishment of infection by the parasite. At first sight this approach appears to be counterintuitive. Indeed, an important requirement of traditional anti-microbial chemotherapy is not to interfere with functions of the host. However, targeting of host discrete components that the parasite seems to modulate has the advantage that drug resistance cannot result from direct alterations of the drug target [12]. Quinolines and artemisinins are exceptional amongst anti-microbial drugs in that they target heme, a component of the host [13]. Resistance to quinolines developed very slowly as it required the selection of variants that affect drug transport. Resistance to artemisinins has not yet developed in the field, although some studies indicate that the parasite may acquire resistance to this drug [12], [14]. Resistance is even less likely to occur if the targeted host component remains outside the pathogen [15].

In the present study, we show that the tyrosine kinase inhibitor genistein inhibits sporozoite development, in the liver *in vitro* as well as *in vivo*. The reduction of liver infection results in a reduction of the parasite load in the subsequent blood stage of the infection. Decreasing the parasite load in the liver has been shown to reduce the severity of the disease in the subsequent blood stage of the infection [2], [3]. Here, we show that genistein reduces the risk of development of severe disease during the later blood stages by reducing the parasite load in the liver. Our data shows that, in the amount included in the supplemented diet, genistein does not affect blood parasitemia directly, which does not imply that, at higher concentrations, genistein does not have a direct effect on malaria blood stages, as in fact previously shown *in vitro* [6]–[8]. If the results described here, using malaria mouse models, can be reproduced in humans, genistein has the potential to decrease malaria morbidity and mortality in endemic areas. Epidemiological studies show that after the initial period in which children are susceptible to severe malaria, there is a sequential development of three types of protective immunity: first, immunity that reduces the incidence of life-threatening disease; second, immunity that reduces the incidence of symptomatic infection; and only then, third, immunity that reduces parasitization [16]. Natural nonsterilizing immunity that develops in people in sub-Saharan Africa occurs by constant exposure to the parasites [17]. Thus, the fact that genistein does not totally block infection might turn out to be an advantage of the strategy of prophylaxis that we envisage. In endemic areas, genistein may reduce the incidence of severe malaria by decreasing liver infection and, at the same time, allow the establishment of natural immunity.

In our previous work we have shown that sporozoite-induced Met phosphorylation facilitated the development of the parasite in the liver [9]. Here we show that the tyrosine kinase inhibitor genistein inhibits sporozoite induced Met phosphorylation. It is conceivable that this inhibition of Met activation is at least partly responsible for the inhibition of parasite development by genistein. However, additional or even alternative mechanisms of action by genistein cannot be excluded at the present time. Recent results from our laboratory have implicated several other kinases, including at least one tyrosine kinase, in the liver stage of *Plasmodium* infection (Prudêncio *et al.*, unpublished results). Thus, genistein may reduce infection by inhibiting more than one tyrosine kinase. Genistein is also known to inhibit a variety of other enzymes such as thyroid peroxidases [18], and topoisomerases [19], [20]. Moreover genistein interacts with P1-purinergic (adenosine) receptor in thyroid cells [21] as well as with several members of the nuclear receptor family such as estrogen receptors and peroxisome proliferator activator receptors (PPARs) [22]–[24]. Whether inhibition of infection by genistein involves any of these and/or other proteins expressed by hepatocytes is not yet known. In the present study we have not observed an effect of genistein pretreatment of sporozoites on the infection rate. This finding suggests that genistein has no direct effect on protein(s) expressed by sporozoites before infection, but does not exclude an effect of genistein on the parasite after infection. Parasite-encoded tyrosine kinases are unlikely to be targets for the action of genistein, since the recent annotation of genomes from several *Plasmodium* species have not identified genes coding for molecules clustering for the tyrosine kinase (TK) group [25]. However, one might speculate that the inhibition of topoisomerase I and II, by genistein, reduced the replication rate of the parasite. While this possibility cannot be excluded, it is unlikely that the treatment used in our study resulted in the high genistein levels in hepatocytes that are required to inhibit topoisomerases [19], [20], [26]–[28]. In addition, the fact that *Plasmodium* blood stages are not affected by the same amounts of genistein (Figure 4D) suggests that, in the concentrations and conditions used, a direct effect of this drug on the parasite is quite unlikely.

The diet of many people in the world, in particular in Japan, is enriched in genistein. Soy beans are a major source of genistein. Because of its putative health benefits (inhibition of osteoporosis, anti-cancer effects, anti-inflammatory effects) genistein is widely available in developed countries as a food supplement. If genistein is to be used for malaria prophylaxis, its safety in neonates, young children and pregnant women is mandatory. The safety of genistein supplementation, and in particular of soy-based infant formulae, has been reviewed in the UK by an expert committee and by an expert pannel of the National Toxicology Program in the USA (http://www.food.gov.uk/science/ouradvisors/toxicity/COTwg/wg_phyt., http://cerhr.niehs.nih.gov/chemicals/genistein-soy/genistein/genistein-eval.html and [29]). Both reports consider soy-based infant formula safe, although both highlight the fact that genistein accounts for only a small percentage of the total of the isoflavones present in soy formulae. While the committee has not found any clear evidence for adverse effects of soy-rich diets in infants and adults, it did emphasize the need for further research on the potential, adverse effects of soy-based infant formulae. Several recent studies have addressed the safety of genistein supplementation in adults. No obvious adverse effects were observed after daily ingestion of up to 54 mg of genistein [30]. Although in the gavage and i.p. distribution of genistein to mice doses of genistein higher than 54 mg/Kg/day per mouse were used, in our *in vivo* experiments with the genistein supplemented diet genistein amounts in sera were never higher than 5 μM (Table 1), which have been recently considered non-toxic values of total genistein [31].

**Table 1:** Genistein concentration in mouse sera (*p<0,05; ***p<0,001)

Genistein Admnistration (oral)	Time of measurement	Genistein Concentration (μM)	Genistein Concentration (ng/ml)
*Single dose Control (H_2_O)*	6 h after gavage	0,42	114,0
		0,06	17,4
*Single dose (200 mg/kg)*	2 h after gavage*	7,07	1910,6
		15,00	4053,2
		13,25	3581,5
		7,23	1952,7
	6 h after gavage	2,29	619,9
		0,57	153,8
		0,32	87,7
		0,45	124,2
*Control Diet*	7–9 weeks old mice	0,11	32,0
		0,09	26,4
		0,19	52,2
		0,21	55,6
		0,19	51,3
		0,47	126,9
		0,39	104,6
		0,15	41,5
		0,14	37,5
		0,13	36,3
		0,28	75,2
		0,13	36,3
		0,13	35,5
		0,17	46,9
Genistei Supplemented-diet (1000 ppm)***	7–9 weeks old mice	3,32	896,6
		1,99	539,1
		1,35	367,0
		2,26	611,5
		3,02	818,0
		1,34	362,7
		3,14	848,5
		3,75	1013,9
		0,20	55,0
		1,11	300,9
		0,84	227,9
		1,37	371,1
		0,68	184,8
		1,16	313,8
		1,16	313,9
		1,42	385,9

Moreover, genistein is inexpensive, easy to preserve and to distribute. One approach to ensure distribution to a large number of people would be to add the compound to flour, a dietary staple in many malaria endemic countries. Alternatively one might envisage including soy plants in agriculture and soy beans in the diet of populations in endemic countries, as has recently been suggested as a strategy to improve both the health and the economy of these populations (http://www.idrc.ca/en/ev-31644-201-1-DO_TOPIC.html). Since genistein is safe and widely consumed as a natural constituent or a supplement in diets of Western populations, testing its potential for malaria prophylaxis in non-human primate models of malaria might be envisaged as an intermediate step towards determining whether the protective effects we have observed in mice can be confirmed in humans.

## Materials and Methods

### Cells and parasites

HepG2, and Hepa1-6 cells (human and mouse hepatoma cell lines, respectively) were maintained in DMEM 10% FCS, 1% Penicillin/Streptomycin and 1 mM glutamine. HepG2 or Hepa1-6 cells (2×10^5^) were seeded in 24-well plates and allowed to adhere for 24 hours before infection. Huh7 cells (human hepatoma cell line) were maintained in RPMI 10% FCS, 1% Penicillin/Streptomycin and 1 mM glutamine. Huh7 cells (1,75×10^5^) were seeded in 24-well plates and allowed to adhere for 24 hours before infection. Green fluorescent protein (GFP)-expressing *P. berghei* ANKA sporozoites [32] were obtained from the dissection of infected *Anopheles stephensi* mosquito salivary glands.

### *In vitro* infection

*P. berghei* ANKA sporozoites (4×10^4^/well) were added to monolayers of Hepa1-6 or HepG2 cells in 24-well plates. At the time of infection (t0) a range of genistein doses were added to the medium. As control, an equivalent volume of DMSO (dimethylsulphoxide) was added. After 24 hours cells were fixed and stained with mouse anti-EEF (exo-erythrocytic forms of the parasite) antibody (2E6) [33], followed by an anti-mouse secondary antibody. In all experiments, genistein was left in the cultures since the time of addition until the cells were fixed for quantification of infection. Infection was quantified by counting the total number of EEFs per coverslip using fluorescence microscopy (magnification 400×). To assess the time window at which genistein has an effect on infection, *P. berghei* ANKA sporozoites (4×10^4^/well) were added to the monolayers of HepG2 cells and genistein was added at different time-points. Pre-treatment of cells was performed by adding genistein to cells for 2 hours followed by wash and addition of sporozoites. The level of infection was measured as described above, 24 hours post-infection with sporozoites. Quantification of the area of 100 individual EEFs of each group was performed using ImageJ 1.36b software. The results are expressed as areas in μm^2^.

### Viability of genistein-treated cells and sporozoites

HepG2 cells (2×10^5^) were seeded in 24-well plates and allowed to adhere for 24 hours before addition of genistein at doses of 25 μM or 250 μM. Twenty four hours later the number of adherent cells was determined in 10 fields per coverslip, in triplicates. The number of adherent cells was also observed 48 hours post genistein treatment. To test whether genistein had a direct effect on the parasite, sporozoites were treated for 30 minutes with 100μM genistein. Control sporozoites were treated with equivalent volume of DMSO. After 30 min of incubation, sporozoites were washed and added to a monolayer of HepG2 cells seeded 24 hours before. Infection was assessed 24 hours later by determining the number of EEFs in each well. Additionally cells were allowed to grow both in the presence of genistein and taxol, a microtubule depolimirizing drugs that causes cell dead, and 24 hours later percentage of cell death was determined by flow cytometry with propidium iodide staining,

### Fluorescence Activated Cell Sorting (FACS) analysis

FACS analysis at 2, 6, 30 and 44 hours after sporozoite addition was performed to determine the percentage of parasite-containing cells and parasite-GFP intensity within infected cells. Cell samples for FACS analysis were processed as previously described [10].

### Mice and genistein administration

C57BL/6 mice were bred and housed in the pathogen-free facilities of the Instituto de Gulbenkian de Ciência following all the EU regulations. The mice were then moved to the Instituto de Medicina Molecular Animal House were all the experiments were performed. All protocols were approved by the IMM Animal Care Committee. Seven to 10 week old male and female C57Bl/6 mice were treated with genistein or vehicle (control), and infected with *P. berghei* ANKA sporozoites. For intraperitoneal administration, mice were treated with a single dose of genistein (200 mg/kg in 200 μL DMSO) or DMSO alone (controls), immediately before *P. berghei* ANKA sporozoite inoculation. For oral administration, a suspension of genistein in water (200 μL, 200 mg/kg) was given to mice by gavage 6 hours prior to infection with 1×10^4^ *P. berghei* ANKA sporozoites. For genistein administration in the diet, mice were given food supplemented with one thousand parts per million (1000 ppm) genistein (genistein 1000 ppm RM3(P)–SDS, England). Control mice were fed with the same diet without genistein supplementation (RM3(P)–SDS, England). Mice were fed starting on their weaning date, and for at least 5 weeks before infection with sporozoites, or breeding couples and their litters were kept on genistein supplemented or control diet through generations. All mice were infected with 1×10^4^ *P. berghei* ANKA sporozoites between the ages of 7 and 9 weeks old and liver infection was determined 40 hours later. In a parallel set of experiments, sporozoite infection was allowed to proceed to blood stage, to assess the impact of genistein in disease progression.

### Quantification of liver infection by quantitative Real Time PCR

Mice treated with genistein and controls were infected by intravenous injection with 1×10^4^ *P. berghei* ANKA sporozoites. Forty hours post-sporozoite injection, livers were dissected and homogenized, and total RNA was extracted with an RNeasy Mini kit Quiagen, according to the manufacturer's recommendation. cDNA was obtained by reverse transcription using the First-strand cDNA synthesis kit (Roche) following the manufacturer's instructions. Quantitative RT-PCR reactions were performed for quantification of parasite load, using an ABI PRISM 7000, Applied Biosystem, and specific primers for *Plasmodium berghei* 18 S rRNA (5′-AAGCATTAAATAAAGC GAATACATCCTTAC-3′ and 5′-GGAGATTGGTTTTGACGTTTATGTG-3′); or specific primers for *P. yoelii* 18 S rRNA (5′ –GGGGATTGGTTTTGACGTTTTTGCG-3′ and 5′-AAGCATTAAATAAAGCGAATACATCCTTAT-3′) were used for quantification of parasite load in the livers of mice 40 hours after sporozoite challenge [34]. The amplification program consisted of incubation at 50°C for 2 min, 95°C for 10 min, followed by 50 cycles at 95°C for 15 s and 60°C for 1 min. For external standardization, plasmids encoding cDNA fragments cloned into TOPO TA, Invitrogen; corresponding to the amplified regions of the corresponding genes, were utilized. Copy numbers were calculated comparing the cycle numbers of the log-linear phase of the samples with the cycle numbers of the external standards.

### Genistein quantification in the sera

“Free” analytes_aglycones, were extracted from the plasma/serum sample with ethyl acetate. The organic phase was evaporated and the dried residue was reconstituted in the mobile phase. For determination of the “total” isoflavones the glucuronide- and sulphate-conjugates were cleaved by enzymatic hydrolysis prior to ethyl acetate extraction. Chromatographic separation was performed by HPLC/MS on a C18-column in the gradient mode. Mass spectrometric detection (Agilent LC/MSD VL single-quadrupole-mass spectrometer equipped with elektrospray-ion source) was carried out with negative electrospray ionisation in the SIM-mode. Specifically, 200 μl of plasma were transferred into a 2 mL Eppendorf tube and 100 μl of enzyme solution were added. For enzymatic cleavage of the glucuronide- and sulphate-conjugates the samples were incubated at 38°C for 3 h, followed by addition of 30 μl internal standard solution. For extraction, 1200 μl ethyl acetate was added to the sample, shaken for 5 min at 300 rpm and centrifuged for 10 min at 10000 rpm and 10°C. 1000 μl of the supernatant was transferred into a 2 ml Eppendorf tube and evaporated at 30–35°C to dryness with a Vortex-Evaporator or Speed Vac. The dried residue was reconstituted in 150 μl mobile phase, briefly vortexed, and transferred into an HPLC vial. Forty to 75 μl were injected onto the analytical column and analyzed by HPLC-MS [35]. Data acquisition of extracted ion chromatograms, integration and quantification was performed with the Chemstation Software (Version 9.03 or higher) from Agilent Technologies.

### Quantification of blood infection and severity of disease

After sporozoite inoculation, peripheral blood parasitemias were followed daily by counting parasites in Giemsa stained thin blood films. To accurately quantify parasitemia in the first 96 hours post-infection, 50 to 100 microscope fields were examined in each blood film. At later time points, when the levels of parasitemia were higher, 20 microscope fields were counted per mouse. Mice were checked daily for development of ECM symptoms, which include ataxia, paralysis, deviation of the head and convulsions, coma and death.

### Blood stage infection

Mice fed on a 1000 ppm genistein supplemented diet or control diet were infected with 1×10^6^ *P. berghei* ANKA iRBC that were freshly obtained after one passage from infection with a frozen vial. Peripheral blood parasitemia and ECM symptoms were determined as described above.

### Whole-cell lysate preparation

To detect the human hepatocyte growth factor receptor (h-MET) and determine its phosphorylation state, total cell extracts were prepared by suspending cell pellets in cold buffer A (20 mM Tris-HCl, pH 7,5; 1% (v/v) NP40; 13,7 mM NaCl; 0,5 mM EDTA; 15% (v/v) glicerol; 1 mM DTT) suplemented with protease and phosphatase inhibitors, for 30 min on ice followed by centrifugation at 14000 rpm, for 15 min at 4°C. Protein concentrations were determined using the Bio-Rad Laboratories DC protein assay kit and equal amounts of protein extracts (40 mg) were separated by 8% SDS-PAGE gels, transferred onto nitrocellulose membranes and processed for Western blotting. The antibody directed to phospho-MET was used at 1∶1000 dilution (#3126; Cell signalling technology, Inc.) and β-chain of human MET was detected with the antibody MET (C-12) at 0,5 μg/mL dilution (#sc-10; Santa Cruz Biotechnology, Inc.). After incubation with the primary antibodies the enhanced chemiluminescence detection system ECL and Kodak films (Amersham Pharmacia Biotech, Piscataway, NJ) were used to visualize proteins on the nitrocellulose blots.
